# Phytochemical analysis and anti-infective potential of fungal endophytes isolated from *Nigella sativa* seeds

**DOI:** 10.1186/s12866-023-03085-4

**Published:** 2023-11-16

**Authors:** Nourhan Hisham Shady, Sara Khalid Sobhy, Yaser A. Mostafa, Ramadan Yahia, Stefanie P. Glaeser, Peter Kämpfer, Mo’men H. El-Katatny, Usama Ramadan Abdelmohsen

**Affiliations:** 1 Department of Pharmacognosy, Faculty of Pharmacy, Deraya University, New Minia City, Minia Egypt; 2 Department of Botany and Microbiology, Faculty of Science, Minia University, Minia, 61519 Egypt; 3 Faculty of Pharmacy, Deraya University, New Minia City, Minia Egypt; 4 Pharmaceutical Organic Chemistry Department, Faculty of Pharmacy, Assiut University, Assiut, 71526 Egypt; 5 Pharmaceutical Chemistry Department, Faculty of Pharmacy, Badr University in Assiut, Assiut, 77771 Egypt; 6 Department of Microbiology and immunology, Faculty of Pharmacy, Deraya University, New Minia City, Minia Egypt; 7 Institute of Applied Microbiology, Justus-Liebig University Gießen, Gießen, Germany; 8 Department of Pharmacognosy, faculty of pharmacy, Minia university, Minia, Egypt

**Keywords:** *Nigella sativa*, Endophytes, Metabolomics, Antimicrobial, Molecular docking

## Abstract

Endophytic fungi, particularly from higher plants have proven to be a rich source of antimicrobial secondary metabolites. The purpose of this study is to examine the antimicrobial potential of three endophytic fungi *Aspergillus* sp. SA1, *Aspergillus* sp. SA2, and *Aspergillus* sp. SA3, cultivated from *Nigella sativa* seeds against *Staphylococcus aureus* (ATCC 9144), *Escherichia coli* (ATCC 25922), *Pseudomonas aeruginosa* (ATCC 27853), *Klebsiella pneumoniae* (ATCC 13883), MRSA (ATCC 33591), and human pathogen *Candida albicans* (ATCC 10231). Furthermore, the most active cultivated endophytic fungi were molecularly identified via internal transcribed spacer (ITS) sequencing. HR-ESIMS guided approach has been used successfully in chemical profiling of 26 known bioactive secondary metabolites (1–26), which belongs to different classes of natural compounds such as polyketides, benzenoids, quinones, alcohols, phenols or alkaloids. Finally, *in-silico* interactions within active site of fungal Cyp51 and bacterial DNA gyrase revealed possibility of being a hit-target for such metabolites as antimicrobials.

## Introduction

Endophytes are fungi, bacteria, and yeasts that live in healthy plant tissues for at least a stage of their life cycles without showing any symptoms of disease or producing a harmful impact on their host plants [1]. Endophytes can survive in plant tissues for a long time and protect the plant from biotic and abiotic stress [2]. Endophytic fungal bioactive metabolites are useful as new drugs due to their diversity of biological activities. A variety of bioactive metabolites in endophytes, including antiviral, anticancer, anti-diabetic, and antibacterial compounds, have been reported [3]. Infectious diseases are a long-lasting and a serious public health problem that affects people all over the world [4]. Today, numerous bacteria, including *Staphylococci, Enterococci, Gonococci, Streptococci*, and others, demonstrate multidrug resistance [5]. There is an urgent need to look for novel compounds for drug development, industry, and agricultural applications [6–9]. Therefore, finding new antibiotics is crucial to combat these resistant bacteria [10]. Natural compounds from endophytic fungi are potential candidates for new antibiotics against pathogenic bacteria [11]. Therefore, in recent years, more attention has been given to the endophytic fungi of medicinal plants, which represent a rich source of new and useful natural compounds of interest to the pharmaceutical and agricultural industries and a potential source for the discovery of novel microorganisms [12–14]. *Nigella Sativa* seeds (Ranunculaceae), commonly known as black seed or black cumin, traditionally used for treatments of various illness affected lungs, kidney, GIT, circulatory and immune systems [15, 16]. The powder of *N. sativa* seeds has antibacterial activity comparable with other antibiotics such as, tetracycline, ampicillin, levofloxacin, gentamycin and streptomycin [17, 18]. In this work, three endophytic fungi have been isolated and identified from *Nigella sativa* (F. Ranunculaceae) seeds growing in Egypt. The isolated fungi were identified morphologically and microscopically up to species to be *Penicillium* sp. SA1, *Aspergillus* sp. SA2, and *Aspergillus* sp. SA3. The isolated fungal strains were cultivated using the OSMAC approach (One Strain many Compounds) method “is a simple and effective approach for activating metabolic pathways and has been successfully applied”, this terms including the alteration in culture conditions resulting in diversity in the microorganisms production and subsequently variation in secondary metabolites. Moreover, the chemical profiles were explored by LC‒MS-based metabolomics. Furthermore, we have investigated these three strains against five bacterial strains (*Staphylococcus aureus, Pseudomonas aeruginosa, Escherichia coli, Klebsiella pneumoniae*, MRSA) and *Candida albicans* to evaluate their antimicrobial activity. *In-silico* studies were performed within crystal structure of two protein, fungal sterol 14α-demethylase and bacterial DNA gyrase.

## Experimental

### Plant material

Fresh plant materials (seeds) were collected from the Agricultural research center in Malawi, EL- Minia, Egypt. Prof. Nasser Barakat (Department of Botany and Microbiology, Faculty of Science, Minia University) identified the investigated plant.

### Isolation and identification of endophytic fungi

Isolation of endophytic fungi from seeds of *Nigella sativa* was carried out using the protocol by Strobel et al. with slight modifications [19].

*Nigella sativa* seeds were collected, and subjected to surface sterilization first by distilled water followed 70% EtOH for 1 min, then distilled sterilized water, 1.0% sodium hypochlorite (NaOCl) (v/v) for 1 min, and finally with sterilized water. *Nigella sativa* seeds were crushed into small particles using sterilized pestle and mortar and homogenized with sterilized water. Each 100 µL of the suspension was spread on the surface of potato dextrose agar medium plate (PDA, 200 g potato, 20 g glucose, and 15 g agar in 1 L distilled water, PH 6.0) supplemented with gentamycin (100 mg/ L) and amoxicillin (100 mg/ L) to suppress bacterial contamination. The plates were then incubated for 21 days in the incubator, and the plates were examined daily during the incubation period and the pure strains of fungi were detected and isolated [20]. The isolated fungal colonies with distinct morphological appearance were isolated, purified and stored in glycerol stock at – 70 ºC for long storage and on a new agar slants at 4 °C for further studies [13]. Endophytes were deposited in the Microbial Repository of Botany and Microbiology (MRBM) Department, Faculty of Science, Minia University, Minia, Egypt, where they were stored at 4 °C. Based on morphological characteristics, out of 20 isolated colonies, three isolates with distinct morphology were selected for further work and named SA1, SA2 and SA3 respectively.

### Molecular identification and phylogenetic analysis

Molecular identification of the most active fungal isolated strains recovered from *Nigella sativa* seeds was achieved by sequence analysis of the fungal internal transcribed spacer (ITS) region including ITS1, the 5.8 S rRNA gene, and ITS2 sequences according to [21]. In brief, DNA was extracted from fungal biomass using the MasterPure Yeast DNA extraction kit (epientre, Madison, Wisconsin). DNA amplification was performed with universal fungal primers NS1 [22] (x) and ITS-4 [23]. Sanger sequencing with primer ITS-4 was performed by LGC Genomics (Berlin, Germany). Manual sequence corrections and phylogenetic analyses were performed with MEGA11 version 11.0.1 [24]. Next related taxa were determined by BLASTn analysis against the ITS RefSeq Targeted Loci project database (BioProject PRJNA177353; update 2023/03/22) provided in the BLASTn tool of the NCBI (https://www.ncbi.nlm.nih.gov/). ITS sequences of next related reference strains were imported into MEGA11 and aligned with ClustalW [25]. All nucleotide positions were considered with uniform rates. The phylogenetic tree was constructed with the maximum likelihood method based on the Kimura 2-parameter model [26]. The phylogeny was tested by the bootstrap method (100 replications). A total of 39 sequences and 620 nucleotide positions were in the final dataset. Two *Penicillium* spp. were included as outgroup sequences. The ITS sequences of *Aspergillus* sp. SA1, *Aspergillus* sp. SA2 and *Aspergillus* sp. SA3 weredeposit in Genbank/EMBL/DDBJ with Accession numbers OQ652078 to OQ652080.

The three fungal strains SA1, SA2 and SA3 were compared by genomic fingerprinting using rep- and RAPD-PCR techniques to determine if the strains are clonal or genetically identical. Analysis was performed according to Glaeser et al. [27] using primer (GTG)5 and two fungal specific primers, GACA (5´- GACAGACAGACAGACA-3´) and NS3 (5´- GCAAGTCTGGTGCCAGCAGCC-3´) [28]. PCRs were performed in a total volume of 15 µl with 1 x DreamTaq buffer, 0.2 mM of each dNTP, 1 µM primer (one per reaction), 0.2 mg/ml bovine serum albumin, 0.02 U/µL DreamDNA polymerase (all chemicals expect primers, Thermo Fisher Scientific, ) and 3 µl cell lysate of fungal biomass. Following cycling conditions were used for GTG5 PCR: 95 °C, 3 min, followed by 30 cycles 94 °C for 30 s, 53 °C for 60 s, 70 °C for 8 min, and finally 70 °C for 16 min and fungal fingerprintings: 95 °C for 3 min, followed by 40 cycles 95 °C for 30 s, 36.5 °C (GACA) and 48.5 °C (NS3) for 30 s, 72 °C for 1 min, and finally 72 °C for 6 min. PCR products were resolved by agarose gel electrophoresis (1.5% agarose, 3.33 V/cm, 150 min) and stained with ethidium bromide.

### Fermentation and extraction

The three fungal endophytes; *Aspergillus* sp. SA1, *Aspergillus* sp. SA2, and *Aspergillus* sp. SA3 (depending on molecular identification) were fermented using the solid approach [29]. In the solid treatment, 150 µL of each strain were inoculated and streaked on ten solid plates (petri dishes : 15 cm )of the media: PDA (200 g potato, 20 g glucose, and 15 g agar in 1 L distilled water). The agar plates were cut into pieces and extracted with 300 mL of ethyl acetate (3 times). Ethyl acetate was then evaporated using rotary evaporator (Heidolph ® 125, 35 °C, 154 rpm) and the yielded dry extract was kept in refrigerator for further analysis.

### LC*-*MS metabolomic analysis

Metabolomic profiling was performed on the crude fungal extracts on an Acquity Ultra Performance Liquid Chromatography system coupled to a Synapt G2 HDMS quadrupole time-of-flight hybrid mass spectrometer (Waters, Milford, CT, USA). Chromatographic separation was carried out on a BEH C18 column (2.1 × 100 mm, 1.7 μm particle size; Waters, Milford, CT, USA) with a guard column (2.1 × 5 mm, 1.7 μm particle size) and a linear binary solvent gradient of 0–100% eluent B over 6 min at a flow rate of 0.3 mL·min^−1^, using 0.1% formic acid in water (v/v) as solvent A and acetonitrile as solvent B. The injection volume was 2.0 µL and the column temperature was 40 ◦C. Ms Converter software was used to convert the raw data into divided positive and negative ionization files. Obtained files were then subjected to the data mining software MZmine 3 (Okinawa Institute of Science and Technology Graduate University, Japan) for deconvolution, peak picking, alignment, deisotoping, and formula prediction. The databases used for the identification of compounds were MarinLit: http://pubs.rsc.org/marinlit/, and Dictionary of Natural Products(DNP)2018: http://dnp.chemnetbase.com/faces/chemical/ChemicalSearch.xhtml [30, 31].

### Antimicrobial activity

The antimicrobial activity of different extracts of *Aspergillus* sp. SA1, *Aspergillus* sp. SA2, and *Aspergillus* sp. SA3 were tested against five pathogenic bacteria *Staphylococcus aureus* (ATCC 9144), *Escherichia coli* (ATCC 25,922), *Pseudomonas aeruginosa* (ATCC 27,853), *Klebsiella pneumonia* (ATCC 13,883), MRSA (ATCC 33,591), and human pathogen *Candida albicans* (ATCC 10,231) using Microtiter bioassay [32]. The dried extracts from each fungus were dissolved in dimethyl sulfoxide (DMSO) to a concentration of 100 mg /mL. Overnight culture in Mueller Hinton broth (Sigma Aldrich, SA) at 37 °C in shaker incubator from each bacterial and the fungal strain was adjusted to 1 McFarland standard which is equivalent to 3.0 × 10^8^ CFU/ mL. First, 100 µL of plain and sterile Mueller Hinton broth was dispensed to all wells of a microtiter plate. 100 µL of the tested extract was added to the first well and serial dilution by 100 µL (1:1) was done from the first well to the 8th well. 100 µL from the last well was discarded. Thus, the concentration of each tested extract was as follow; 50, 25, 12.5, 6.25, 3.125, 1.5625, 0.78125 and finally 0.3906 mg / mL. 5 µL of the bacterial (or fungal) suspension were added to all wells of a microtiter plate except for raw for sterility control. Ciprofloxacin antibiotic was used as positive control for all bacterial strains [33] with the same concentrations as the tested extracts. Fluconazole antifungal agent was used as positive control for the fungal strain [34, 35] with the same concentrations as the tested extracts. Raw contain only the plain sterile medium and the microorganism was used as negative control. The microtiter plates were incubated at 37 ºC for 24 h. After incubation, the plate was measured by ELISA reader at a wavelength of 570 nm using an ELISA plate reader.

### *In-Silico* molecular docking studies

Molecular Orbital Environment (MOE®) software package was used for running molecular docking simulations. The dereplicated identified compounds 1–26 (SA1:7), (SA2:10), (SA3:9) were drawn using ChemDraw®Ultra (ver. 8, 2013) and their energy were minimized using MMFF94x Forcefield energy minimization capability of MOE software with a gradient RMS of 0.0001 kcal/mol and prepared ligands were saved as Microsoft Access Data Base (mdb) file. Crystal structures of two target proteins: Fungal sterol 14a-demthyalse (CYP51; PDB ID: 1EA1; Cytochrome P450 14 alpha-sterol demethylase (CYP51) from Mycobacterium tuberculosis in complex with fluconazole) and bacterial DNA gyrase (topoisomerase II; PDB ID: 5BTC; Crystal structure of a topoisomerase II complex with ciprofloxacin) were downloaded from RCSB Protein Data Bank website (https://www.rcsb.org/ ). Both proteins were prepared in MOE program using protein Quick prep capability to protonate their structures and removal of water molecules. Validation of prepared proteins was performed by docking their co-crystallized ligands (Fluconazole for 1EA1 and Ciprofloxacin for 5BTC) and their docking score (S; Kcal/mol) and RMSD (Å) were in-agreement to reported ones (Informatics in Medicine Unlocked 26 (2021) 100,748 and www.pnas.org/cgi/doi/10.1073/pnas.1525047113. ). The prepared ligands were docked into active site using MOE Alpha triangle placement method and refinements were done by Forcefield, scored using affinity δG (S; Kcal/mol) scoring system.

## Results and discussion

**Fig. 1:**
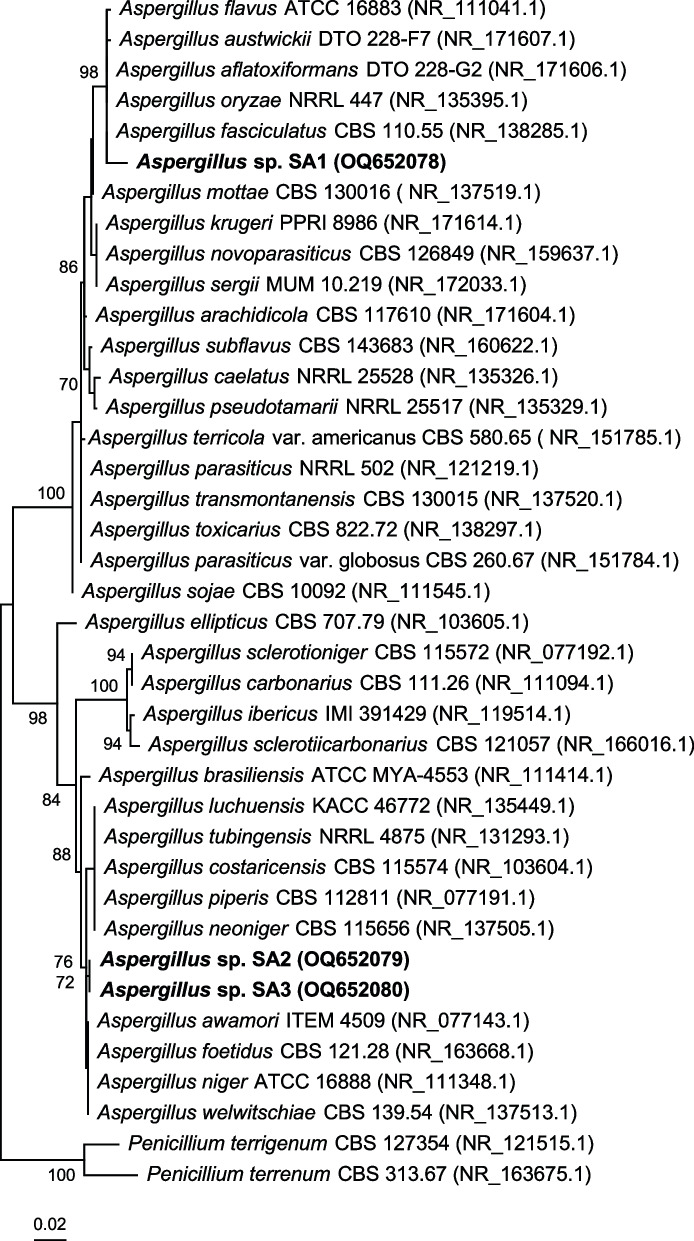
Phylogenetic placement of the three studied fungi *Aspergillus* sp. SA1, *Aspergillus* sp. SA2 and *Aspergillus* sp. SA3 based on the fungal ITS sequence region ITS1–5.8 S – ITS2. The tree was constructed with the maximum-likelihood method in MEGA11. In total 39 sequences and 620 nucleotide positions were considered by the analysis. Numbers ad nodes represent bootstrap values of 70% and above. Two *Penicillium* sp. strains were used as outgroup. Bar: 0.02 substitutions per nucleotide position

**Fig. 2:**
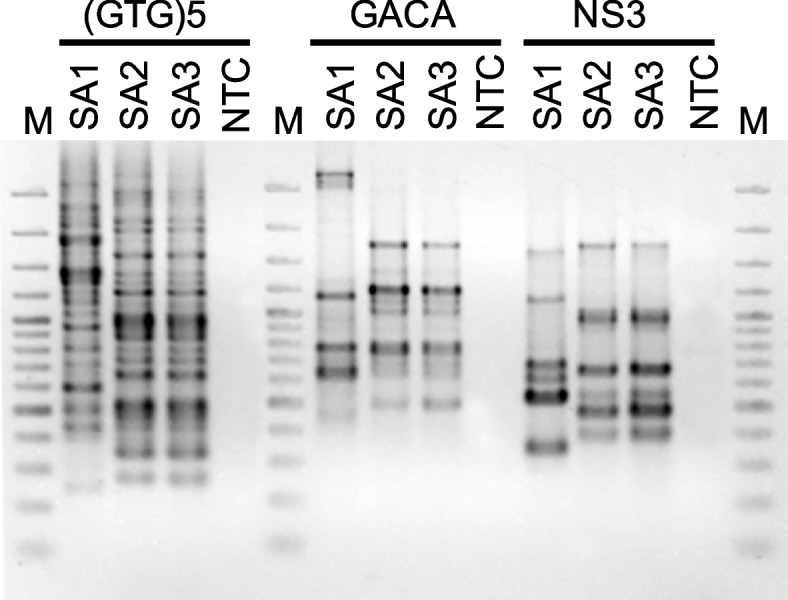
Comparative genomic fingerprint analysis of strains *Aspergillus* sp. SA1, *Aspergillus* sp. SA2 and *Aspergillus* sp. SA3. Analyses were performed with primers (GTG)5, GACA and NS3. Depicted are fingerprint patterns resolved on a 1.4% agarose gel after staining with ethidium bromide. NTC: No template control; M: 100 bp DNA marker

Until now, various reports have highlighted the isolation, identification, and biological properties of different phytoconstituents produced by medicinal plants, although few studies have referred to those provided by their endophytic fungi. Three different fungal strain coded as SA1, SA2, and SA3 were isolated and identified morphologically in additionally to phylogenetic analysis from sterilized seeds of *Nigella sativa* plant their names (*Aspergillus* sp. SA1, *Aspergillus* sp. SA2, and *Aspergillus* sp. SA3). Strain SA1 shared with 99.79% highest ITS sequence similarity with *Aspergillus fasciculatus* CBS 110.55 (NR_138285.1) (*Aspergillus* section Flavi). Strains SA2 and SA3 (identical ITS sequences) shared with 99.31% highest ITS sequence similarity to *Aspergillus awamori* ATCC 16,877 (NR_077143.1) and *Aspergillus foetidus* CBS 121.28 (NR_163668.1) (*Aspergillus* section Niger). The phylogenetic placement is shown in Fig. 1. Beside identical ITS sequences strains SA2 and SA3 also shared identical genomic fingerprint patterns (Fig. 2) which indicated genetic clonality or at least a close genetic relationship of the two strains. Strain SA1 showed fitting the phylogenetic differences based on the ITS sequences different genomic fingerprint patterns compared to strains SA2 and SA3.

The three strains were fermented using different media to investigate the chemical diversity of *Aspergillus* sp. SA1, *Aspergillus* sp. SA2, and *Aspergillus* sp. SA3.

**Fig. 3:**
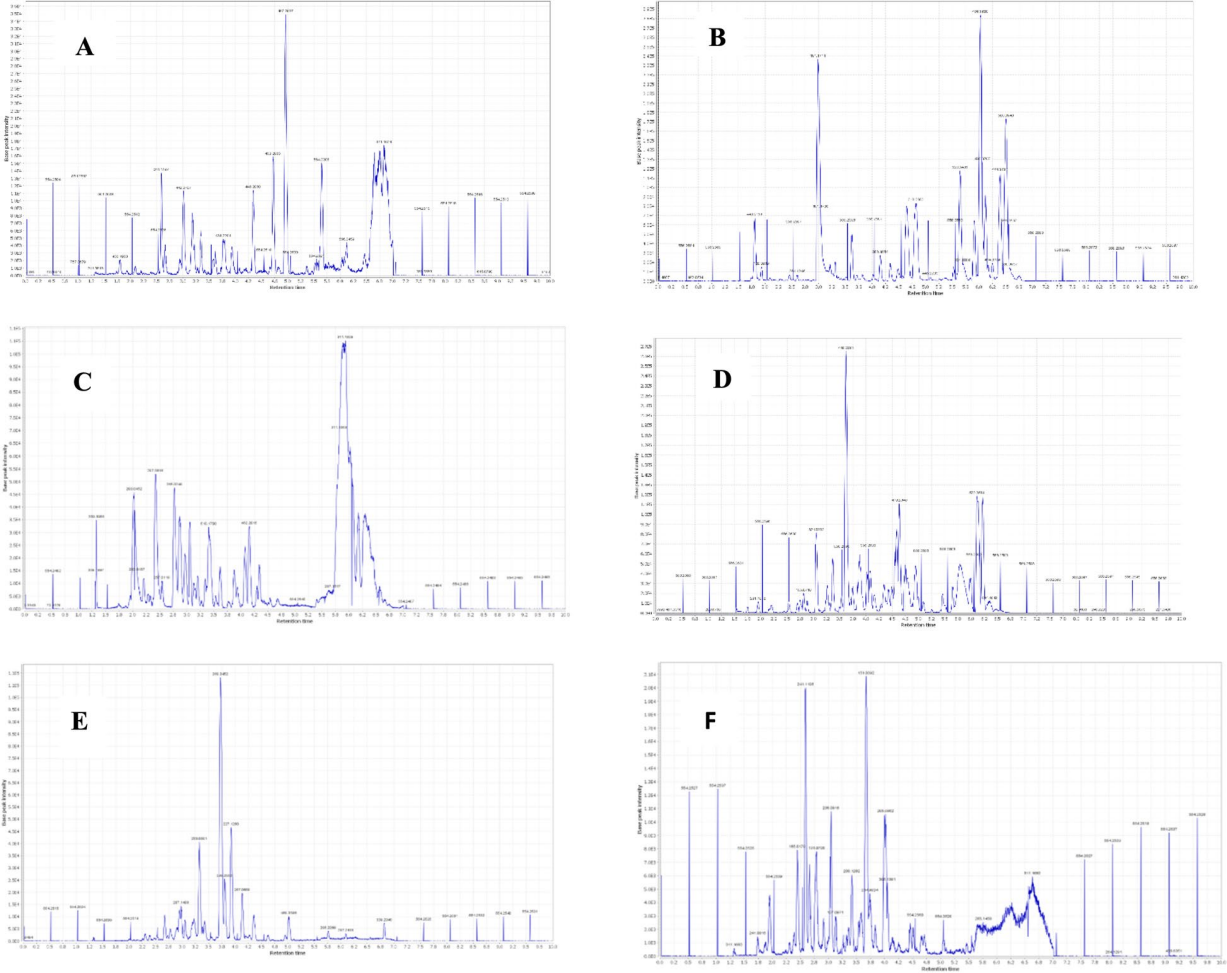
Total ion chromatograms of the crude extracts **A**: *Aspergillus* sp. SA1 (Negative mode). **B ***Aspergillus* sp. SA1 (positive mode). **C** *Aspergillus* sp. SA2 (Negative mode). **D ***Aspergillus* sp. SA2 (positive mode). **E ***Aspergillus* sp. SA3 (Negative mode). **F ***Aspergillus* sp. SA3 (positive mode)

**Fig. 4:**
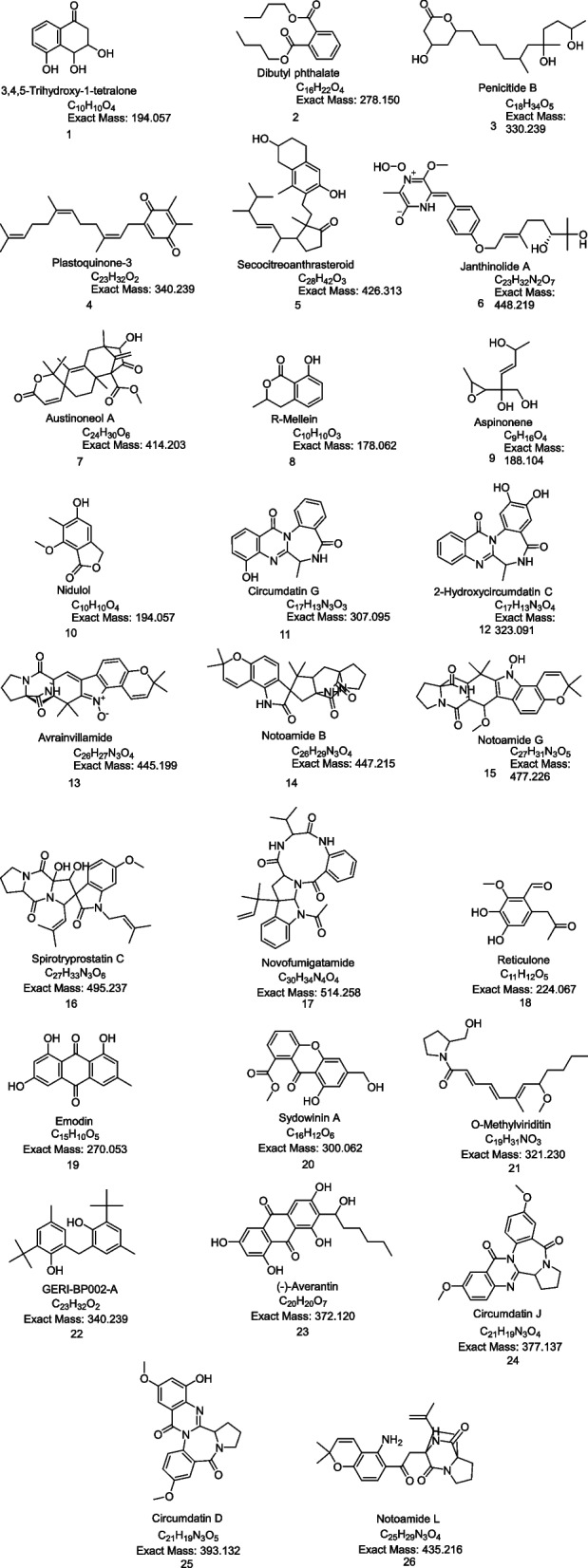
Chemical structures of the annotated identified compounds from the extracts of *Aspergillus* sp. SA1, *Aspergillus* sp. SA2, and *Aspergillus* sp. SA3 isolated from *Nigella sativa*

The crude culture extracts were subjected to high-resolution mass spectrometry (HR-MS) analysis. Different metabolites produced by the three strains under different culture conditions were explored by LC‒MS-based metabolomics analysis as shown in the total ion chromatograms Fig. 3. Metabolic profiling was carried out for all extracts of *Aspergillus* sp. SA1, *Aspergillus* sp. SA2, and *Aspergillus* sp. SA3 results in annotation of 26 compounds (1–7), (8–17) and (18–26) respectively, as shown in Table 1; Fig. 4. Employing macros and algorithms that coupled MZmine with online and internal databases, such as MarinLit, DNP, and METLIN, as well as the comparison with the given literature data, the compounds were annotated. Many other chemical classes of metabolites were dereplicated such as polyketides, benzenoids, quinones, alcohols, phenols, and alkaloids. Identified compounds from *Aspergillus* sp. SA1 were shown in Table 1; Fig. 4 including Dibutyl phthalate (2) that was dereplicated from the mass ion peak at *m/z* 278.150 in agreement with the molecular formula C_16_H_22_O_4_, was previously isolated for the first time from this plant source have significant activity against *Klebsiella pneumonia* and *Pseudomonas aeruginosa*. The in vitro antibacterial evaluation of dibutyl phthalate forms a primary platform for further phytochemical and pharmacological studies [36]. Moreover, the mass ion peak at *m/z* 330.239, consistent with the molecular formula C_18_H_34_O_5_, was also identified as Penicitide B (3); Penicitide B as antifungal agent, [37, 38]. In the same vein, the mass ion peak at *m/z* 340.239, consistent with the molecular formula C_23_H_32_O_2_ was annotated as Plastoquinone-3 (4); was detected in spinach chloroplasts [39]. Similarly, Janthinolide A (6), which showed the molecular formula C_23_H_32_N_2_O_7_ and was annotated from the mass ion peak at *m/z* 448.219, these compound isolated from the coral endophytic *Penicillium janthinellum* [40], exhibited antifungal properties [41]. Another compound with the molecular formula C_24_H_30_O_6_ was identified as Austinoneol A (7) based on the observed mass ion peak at *m/z* 414.203, was previously isolated from *Penicillium* sp. [42]. On the other hand, metabolic profiling of the crude extracts of *Aspergillus* sp. (SA2) revealed a variety of metabolites as shown in (Table 1; Fig. 3), of which the mass ion peak at *m/z* 178.062 in consonance with the molecular formula C_10_H_10_O_3_ was also annotated as R-Mellein (8). This compound belongs to the family of pentaketides and have a widely distributed dihydroisocoumarin derivative in fungi [43], was first isolated in 1933 from *Aspergillus melleus* and exhibited a strong fungicidal agent [44]. Moreover, the mass ion peak at *m/z* 188.104, consistent with the molecular formula C_9_H_16_O_4_, was also identified as Aspinonene (9). This compound belongs to pentaketides previously purified from *Aspergillus ochraceus* (DSM-7428) in 1997 pentaketides [45]. When screened for activity towards MRSA aspinonene displayed mild inhibitory properties. Another new alkaloid from the culture broth of *Aspergillus ochraceus* with the molecular formula C_17_H_13_N_3_O_3_ was characterized as circumdatin G (11) based on the observed mass ion peak at *m/z* 307.095. This compound showed biological activity it inhibit the final stage of polyprotein processing during hepatitis C virus replication [46]. Moreover, an alkaloid with the molecular formula C_17_H_13_N_3_O_4_ was characterized as 2-hydroxycircumdatin C (12) based on the observed mass ion peak at *m/z* 323.090. This compound was also formerly identified from a natural source for the first time [47]. This compound isolated from *A. ochraceus* and showed notable antioxidant activity. The mass ion peak at *m/z* 445.199 in consonance with the molecular formula C_26_H_27_N_3_O_4_ was also annotated as avrainvillamide (13), was first isolated in 2000 from the marine fungus *Aspergillus* sp. [48], showed anti-insecticidal and antibacterial properties. This compound was also have antibiotic activity that inhibits the growth of multi-drug-resistant *Staphylococcus aureus*, *Streptococcus pyogenes*, and *Enterococcus faecalis* [49]. Additionally, notoamide B (14), dereplicated from the mass ion peak at *m/z* 447.215 and the corresponding molecular formula C_26_H_29_N_3_O_4_, also was previously identified as a metabolite of *Aspergillus* sp., showing stronger antibacterial activity against *E. coli* and *P. aeruginosa* also have potent insecticidal activities against H. *armigera* [50]. Furthermore, The mass ion peak at *m/z* 495.237 in consonance with the molecular formula C_27_H_33_N_3_O_6_ was also annotated as Spirotryprostatin C (16), was isolated from *Aspergillus fumigatus* [51]. This compound showed significant antibacterial activity against certain microbial pathogens, in which the highest antibacterial activity against *Escherichia coli*, *Acinetobacter baumannii*, *Pseudomonas aeruginosa, Klebsiella pneumoniae*, methicillin-resistant *Staphylococcus aureus* (MRSA), and *Enterococcus faecalis* [52]. The mass ion peak at *m/z* 514.258 in consonance with the molecular formula C_30_H_34_N_4_O_4_ was also annotated as Novofumigatamide (17), which was formerly identified from *Aspergillus novofumigatus* CBS11520 as the new *Aspergillus* sp. [53]. In the same vein, metabolic profiling of the crude extracts of *Aspergillus* sp.(SA3) revealed a moderate number of metabolites (Table 1; Fig. 3), peak at *m/z* 270.052, consistent with the molecular formula C_15_H_10_O_5_ was annotated as Emodin (19); This compound is an active ingredient of herbal medicine, Emodin (1,3,8-trihydroxy-6-methylanthraquinone) also has been reported to exhibit anti-inflammatory properties by reduction of cytokine production in human T-lymphocytes and endothelial cells [54]. This anthraquinone have antibacterial effects against *Escherichia coli* were proposed to be mediated through inhibition of respiration-driven solute transport in membrane [55]. Likewise, the mass ion peak at *m/z* 300.062 was annotated as Sydowinin A (20); a dimeric isocoumarin with the molecular formula C_16_H_12_O_6_, Sydowinin A is commonly produced by from *Aspergillus sydowi* [56]. Previously reported analogues showed antibacterial activities against common bacteria *Bacillus subtilis* and *Escherichia coli* [57] as well as against the Gram-positive bacterial strains *Mycobacterium smegmatis* ATCC 607 and *Staphylococcus aureus* ATCC 25,923, Gram-negative *Pseudomonas aeruginosa* ATCC 9027, and fungus *Candida albicans* ATCC10231 [58]. The mass ion peak at *m/z* 340.239 in consonance with the molecular formula C_23_H_32_O_2_ was also annotated as GERI-BP002-A (22), which was isolated from culture broth of *Aspergillus fumigatus* F93 by acetone extraction. These novel compound have inhibited ACAT activity by 50% at the concentration of 50 µM in an enzyme assay system using rat liver, GERI-BP002-A is an attractive target for treatment of hypercholesterolemia and atherosclerosis [59]. Additionally, (-)-Averantin (23), dereplicated from the mass ion peak at *m/z* 372.120 and the corresponding molecular formula C_20_H_20_O_7_, was also formerly isolated from *Aspergillus versicolor*, a sponge-derived fungus showing antibacterial activity against several clinical isolates of Gram + strains [60] and exhibited antifungal activities [61]. Moreover, the mass ion peak at *m/z* 377.137, consistent with the molecular formula C_21_H_19_N_3_O_4_, was also identified as Circumdatin J (24); benzodiazepine alkaloids isolated from the fungal strain *Aspergillus ostianus* IMBC-NMTP03, which also revealed wide antimicrobial potential against *Enterococcus faecalis* and *Candida albicans* [62]. The mass ion peak at *m/z* 393.132 in consonance with the molecular formula C_21_H_19_N_3_O_5_ was also annotated as circumdatin D (25), which was originally reported from *Aspergillus ochraceus* [62], this compound significantly inhibit *Enterococcus faecalis* growth and this compound showed antimicrobial effects against the yeast *Candida albicans* [62]. Finally, the mass ion peak at *m/z* 435.216, consistent with the molecular formula C_25_H_29_N_3_O_4_, was also identified as Notoamide L (26); this compound was isolated from *Aspergillus* species [63]. This provoked us to study the antibacterial potential of the total ethyl acetate extracts of the three endophytic fungi isolated from *Nigella sativa* seeds. *Penicillium* sp. SA1, *Aspergillus* sp. SA2, and *Aspergillus* sp. SA3 against five different pathogenic bacteria *Staphylococcus aureus* (ATCC 9144), *Escherichia coli* (ATCC 25922), *Pseudomonas aeruginosa* (ATCC 27,853), *Klebsiella pneumoniae* (ATCC 13883), MRSA (ATCC 33591), and human pathogen *Candida albicans* (ATCC 10,231) using the quantitative antibacterial assay by IC_50_. Overall, the tested samples revealed varying in vitro growth inhibitory potencies against *Staphylococcus aureus, Pseudomonas aeruginosa, Klebsiella pneumoniae*, MRSA pathogenic bacteria, showing IC_50_ values in the range of 0.7‒27.3 µg/mL. As shown in Table 2, the extract of *Penicillium* sp. SA1 cultured on PDA media exhibited the highest antibacterial activity against *Pseudomonas aeruginosa* and *Staphylococcus aureus*, with IC_50_ value of 0.77 and 7.18 µg/mL, respectively, although it has weak activity against *Klebsiella pneumoniae, Escherichia coli*. Likewise, the extract of *Aspergillus* sp. SA2 cultured on the PDA medium, showed the highest activities against *Pseudomonas aeruginosa* and *Klebsiella pneumonia*, with IC_50_ values of 2.5, 27.3 µg/mL but has weak activity against *Staphylococcus aureus, Escherichia coli* and MRSA. In contrast, the extract of *Aspergillus* sp. SA3 cultured on PDA medium show highest activity against MRSA, *Klebsiella pneumonia, Staphylococcus aureus* and *Pseudomonas aeruginosa* with IC_50_ values of 2.5, 2.7, 4.1 and 5.4 µg/mL respectively, while it has weak activity with *Escherichia coli*. However, all the above-mentioned extracts showed higher antimicrobial and antifungal potential as compared to the positive control, Ciprofloxacin that revealed IC_50_ values of 1.2, 11.9, 16.3, 19.6 and 25.7 µg/mL in the case of *Pseudomonas aeruginosa*, *Staphylococcus aureus*, *Escherichia coli*, *Klebsiella pneumonia* and MRSA, respectively (Table 2).

**Table 1:** A list of the annotated metabolites from the investigated extracts of *Aspergillus* sp. SA1, *Aspergillus* sp. SA2, and *Aspergillus* sp. SA3

No.	RT	Exact mass	Calculated mass/ Theoretical mass	Molecular formula	Name	Reference
***Aspergillus*** ** sp. SA1**						
1	3.4	194.057	u194.057	C_10_H_10_O_4_	3,4,5-Trihydroxy-1-tetralone	[64]
2	5.6	278.150	278.151	C_16_H_22_O_4_	Dibutyl phthalate	[36]
3	4.1	330.239	330.240	C_18_H_34_O_5_	Penicitide B	[38]
4	6.8	340.239	340.240	C_23_H_32_O_2_	Plastoquinone-3	[65]
5	6.5	426.313	426.313	C_28_H_42_O_3_	Secocitreoanthrasteroid	[66]
6	4.1	448.219	448.220	C_23_H_32_N_2_O_7_	Janthinolide A	[67]
7	4.9	414.203	414.204	C_24_H_30_O_6_	Austinoneol A	[68]
***Aspergillus*** **sp. SA2**						
8	2.7	178.062	178.062	C_10_H_10_O_3_	R-Mellein	[69]
9	3.1	188.104	188.104	C_9_H_16_O_4_	Aspinonene	[43]
10	3.4	194.057	194.057	C_10_H_10_O_4_	Nidulol	[70]
11	3.3	307.095	307.095	C_17_H_13_N_3_O_3_	circumdatin G	[63] [46]
12	3.0	323.090	323.090	C_17_H_13_N_3_O_4_	2-hydroxycircumdatin C	[63]
13	3.7	445.199	445.200	C_26_H_27_N_3_O_4_	Avrainvillamide	[48]
14	4.3	447.215	447.215	C_26_H_29_N_3_O_4_	notoamide B	[50]
15	4.6	477.226	477.226	C_27_H_31_N_3_O_5_	notoamide G	[71]
16	4.5	495.237	495.236	C_27_H_33_N_3_O_6_	Spirotryprostatin C	[72]
17	3.6	514.258	514.258	C_30_H_34_N_4_O_4_	Novofumigatamide	[53]
***Aspergillus*** **sp. SA3**						
18	2.9	224.067	224.068	C_11_H_12_O_5_	Reticulone	[73]
19	3.6	270.052	270.052	C_15_H_10_O_5_	Emodin	[54]
20	3.7	300.062	300.063	C_16_H_12_O_6_	Sydowinin A	[58]
21	6.2	321.230	321.230	C_19_H_31_NO_3_	O-Methylviriditin	[74]
22	6.8	340.239	340.240	C_23_H_32_O	GERI-BP002-A	[59]
23	3.3	372.120	372.120	C_20_H_20_O_7_	(-)-Averantin	[60]
24	4.4	377.137	377.137	C_21_H_19_N_3_O_4_	Circumdatin J	[62]
25	4.0	393.132	393.132	C_21_H_19_N_3_O_5_	circumdatin D	[75]
26	4.6	435.216	435.215	C_25_H_29_N_3_O_4_	Notoamide L	[63]

**Table 2:** Antimicrobial activities of different ethyl acetate extracts of *Aspergillus* sp. SA1, *Aspergillus* sp. SA2, and *Aspergillus* sp. SA3

Code	Sample	Antimicrobial activity of extracts against the tested microbes					
		IC_50_ ug/mL					
		*Staphylococcus* *aureus*	*Pseudomonas aeruginosa*	*Escherichia coli*	*Klebsiella pneumoniae*	MRSA	*Candida* *albicans*
SA1	***Aspergillus*** **sp.**	7.2	0.8	296.1	437.5	ND	92.5
SA2	***Aspergillus*** **sp.**	49.5	2.6	43.9	27.4	81.5	120.9
SA3	***Aspergillus*** **sp.**	4	5.5	184.4	2.7	2.5	218.1
**Ciprofloxacin**		11.9	1.2	16.3	19.7	25.7	-
**Fluconazole**	-	-	-	-	-	-	1.8

Based on promising results obtained with various fungal extracts on six bacterial and fungal species, we decided to explore their cellular targets virtually using Molecular Orbital Environment (MOE^®^) Software. Molecular docking simulations were performed within active sites of 2 crystal structures obtained from Protein Data Bank (RSCB PDB): Fungal sterol 14α-demethylase (CYP51: a member of Cytochrome P450 superfamily) and bacterial DNA gyrase (bacterial topoisomerase II). Interestingly, most of three extracts’ compounds showed better docking score (S; Kcal/mol) than co-crystallized ligand; Fluconazole (as listed in Table 3).

**Table 3:** Molecular docking of *Aspergillus* sp. SA1, *Aspergillus* sp. SA2, and *Aspergillus* sp. SA3 compound within fungal sterol CYP51 (PDB ID: 1EA1) and bacterial DNA gyrase (PDB ID: 5BTC)

#	Molecule	Fungal Cyp51 (1EA1)		Bacterial DNA Gyrase (5BTC)	
		S (Kcal/mol)	RMSD (Å)	S (Kcal/mol)	RMSD (Å)
Ref.	Fluconazole	-5.34	1.77	NA	NA
	Ciprofloxacin (CFP)	NA	NA	-12.87	0.96
***Aspergillus*** **sp**. **SA1**					
1	3,4,5-Trihydroxy-1-tetralone	-4.38	1.62	-2.68	1.04
2	Dibutyl phthalate	-4.89	1.19	-7.00	1.49
3	Penicitide B	-5.88	1.45	0	0
4	Plastoquinone-3	-7.28	0.83	-3.04	1.47
5	Secocitreoanthrasteroid	-5.60	1.31	-2.79	2.36
6	Janthinolide A	-6.92	1.45	-0.76	2.45
7	Austinoneol A	-5.91	0.58	0	0
***Aspergillus*** **sp. SA2**					
8	R-Mellein	-4.69	1.38	-6.15	1.34
9	Aspinonene	-4.87	1.59	-3.69	1.10
10	Nidulol	-4.64	1.49	-4.39	0.97
11	Circumdatin G	-5.43	1.69	-4.43	2.29
12	2-Hydroxycircumdatin C	-5.67	1.62	-3.77	1.08
13	Avrainvillamide	NA	NA	NA	NA
14	Notoamide B	-6.43	0.66	0	0
15	Notoamide G	NA	NA	NA	NA
16	Spirotryprostatin C	-7.51	1.09	-4.61	2.52
17	Novofumigatamide	-5.77	0.98	0	0
***Aspergillus*** **sp**. **SA3**					
18	Reticulone	-5.29	0.55	-4.94	1.41
19	Emodin	-5.60	0.94	-6.41	0.86
20	Sydowinin A	-6.30	0.89	-7.79	0.99
21	O-Methylviriditin	-5.65	1.11	-8.97	2.05
22	GERI-BP002-A	-7.19	1.06	-5.39	1.30
23	(-)-Averantin	-6.24	1.16	-5.92	1.47
24	Circumdatin J	-6.53	1.25	-4.76	0.62
25	Circumdatin D	-5.77	0.64	-5.05	1.14
26	Notoamide L	NA	NA	NA	NA

**Fig. 5:**
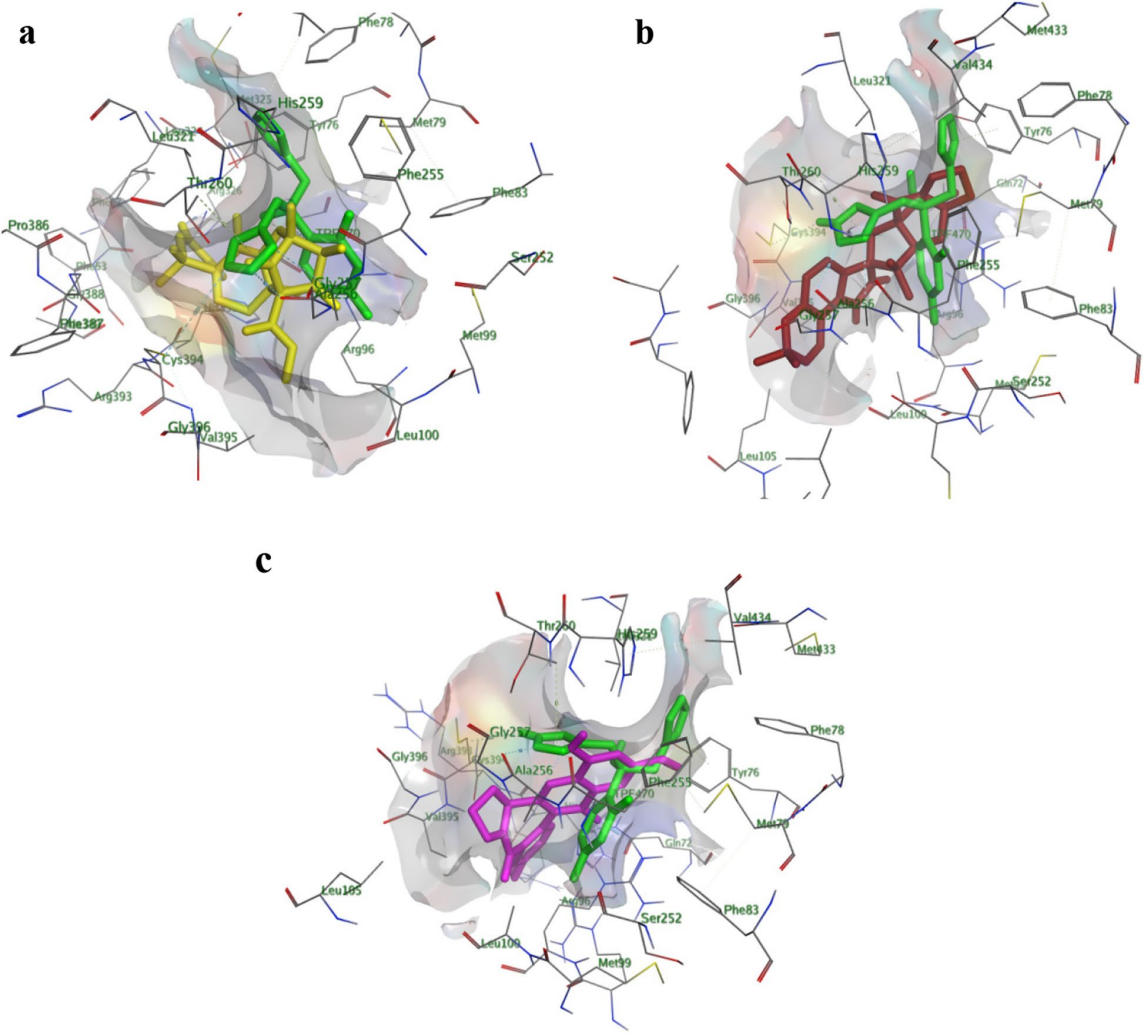
3D Interaction Diagrams of Compounds 7 (Fig.: Yellow-colored), 14 (Fig. **b** Red-colored, and 25 (Fig. **c** Purple-colored) within CYP51 (PDB ID: 1EA1) active site showing also their overlapping with co-crystallized ligand (Fluconazole; Green-colored)

**Fig. 6:**
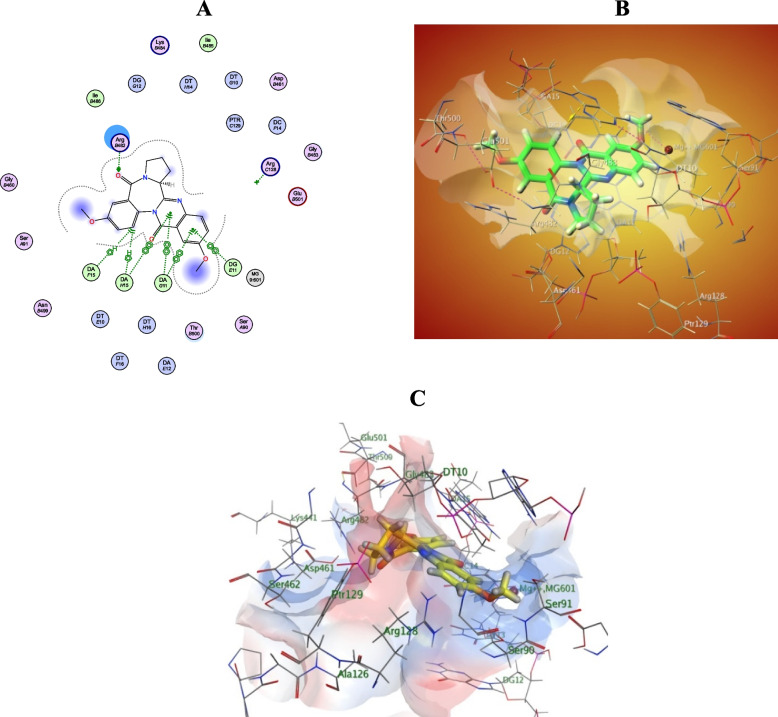
**a** Schematic 2D interaction diagram of Circumatidin J (compound 24) within bacterial DNA gyrase (PDB ID: 5BTC) showing both H-bonding, Bi-Bi, and H-Bi interactions; **b** 3D representation of compound 24 within pocket of DNA gyrase active site showing its close distance with amino acid residues; **c** Electrostatic potential map of DNA gyrase active site and compound 24 functional groups impeded correctly within different potential zones of active site

In addition, these compounds exhibited close contact interactions with amino acid residues lining active site of CYP51, which indicated by their low RMSD values. Compounds 7, 14 and 25 were further explored for their in-site interactions, Fig. 5. The three compounds showed good overlapping with Fluconazole within CYP51 active site, also they showed number of H-bond and H-Bi interactions with various amino acid residues lining active site. Taking the average of docking score (S) for all compounds of each extract, we found that: SA1 (Compounds 1–7) showed the highest docking score with lowest RMSD value over other two extracts, which indeed matches its biological effect on *Candida albicans* isolates and this is a strong point for further investigation of such metabolites. On the other hand, working on the molecular docking simulations within bacterial DNA gyrase active site showed the following interesting observations: generally, molecules of the three extracts showed weak to moderate docking score (S) and RMSD (Å) values, as listed in Table 3). Furtherly, exploration of the binding interactions of Circumdatin J (Compound 24) within DNA gyrase (Fig. 6) with various amino acid residues lining active site, Fig. 5. As seen in Fig. 5a that compound 24 showed many interactions within DNA gyrase active site especially with Arg 482 residue which is one of crucial interactions shown to cause marked inhibition of DNA gyrase enzyme (www.pnas.org/cgi/doi/10.1073/pnas.1525047113.). Additionally, close view of DNA gyrase pocket (Fig. 5b) showed close distance of compound 24 to various amino acid residues lining active site. Finally, electrostatic potential map of DNA gyrase active site (Fig. 5c) showed perfect alignment of various functional groups of compounds 24 with different potential zones of H-donor, acceptor and Van der Waals interactions. Docking poses of ligands with best docking score and/or lowest RMSD values were inspected for their binding interactions for compounds 7, 14, 24, and 25. The above-mentioned results showed the potent effect of the three endophytic fungi (*Aspergillus* sp. SA1, *Aspergillus* sp. SA2, and *Aspergillus* sp. SA3) isolated from *Nigella sativa* seeds, as a powerful source of natural compounds with antibacterial activity against Gram-negative and Gram-positive bacteria. Especially, compounds identified from *Aspergillus* sp. SA3 showed the best average docking score within all the three investigated strains, and this provoked us in the upcoming work, to isolate the most active compounds to evaluate their antimicrobial and antifungal potential via in vitro studies.

## Conclusion

Out of twenty isolated colonies, three isolates with distinct morphology were selected for further work and named SA1, SA2 and SA3 respectively. The three most active endophytic fungi were isolated from *Nigella sativa* seeds, *Aspergillus* sp. SA1, *Aspergillus* sp. SA2, and *Aspergillus* sp. SA3. These endophytic fungi have been shown to be a powerful source of natural compounds with biological activities. Particularly, when it was fermented using PDA culture medium, which could be attributed to its bioactive metabolites, of which twenty-six compounds were identified by LC-HR-ESI-MS belonging to various chemical classes. Crude extracts of endophytic *Aspergillus* sp. SA1, *Aspergillus* sp. SA2, and *Aspergillus* sp. SA3 have shown promising antibacterial activity against Gram-negative and Gram-positive bacteria. Collectively, compounds identified from *Aspergillus* sp. SA3 showed the best average docking score within all the three investigated strains. This could be used as explanatory for remarkable potency of SA3 extract on the five different bacterial species used in this study

## Data Availability

All data generated or analyzed during this study are included in this published article (and its supplementary information files).
